# Effect of HLA on development of asthma

**DOI:** 10.1016/j.amsu.2018.10.003

**Published:** 2018-10-06

**Authors:** Batool Mutar Mahdi, Arwa Tahrir Ramadhan Al-Hadithi, Hyam Raouf, Haider Hashim Zalzala, Laheeb Ali Abid, Zena Nehad

**Affiliations:** a HLA research unit, Al-Kindy College of Medicine, University of Baghdad, Baghdad, Iraq; b Department of Microbiology, Al-Kindy College of Medicine, University of Baghdad, Baghdad, Iraq

**Keywords:** HLA, Asthma, Genetic

## Abstract

**Background:**

Asthma is a disease characterized by a chronic inflammation of the airways caused by the interaction of genetic susceptibility with environmental factors. Inflammation and remodeling are critical components of asthma. It is shown that many genes are involved in the pathogenesis of asthma.

**Aim of study:**

To identify/compare the association between HLA-DRB1 alleles and development of asthma in a sample of Iraqi Arab Muslims.

**Patients and methods:**

A case-control study (forty patients and forty healthy control) was carried out in Medical city Teaching Hospital, Baghdad-Iraq. The study participants were patients with asthma consulted the hospital from September - 2013 to January - 2015. HLA –DRB1genotyping was done using a panel of sequence-specific oligonucleotide probes (SSOP) using HLA-DRB1amplification and hybridization kits (SSO HLA type DRB1 plus and Mastermix for HLA type DRB1 Amp plus kits -Innogenetics-Belgium) using automated method by AutoLipa – 48Innogenetics-Belgum.

**Results:**

There was an increased frequency of HLADRB1*03:01:01 in control group compared with patients group (P = 0.009, Odds ratio = 0.1228, 95% CI: 0.0254–0.5930). Other allele like HLA-DRB1* 070101was significantly increased in asthmatic patients in compares with control group (P = 0.005, Odds ratio = 6.641, 95% CI: 1.7319–25.4657).

**Conclusions:**

HLA alleles have an effect on development asthma in patients with HLA-DRB1*070101 while HLADRB1* 030101 may have a protective effect in Iraqi Arab Muslims individuals against development of asthma.

## Highlights

• Asthma is a disease characterized by a chronic inflammation of the airways caused by the interaction of genetic susceptibility with environmental factors.
• To identify/compare the association between HLA-DRB1 alleles and development of asthma in a sample of Iraqi Arab Muslims.
• A case-control study ( forty patients and forty healthy control) was carried out in Medical city Teaching Hospital, Baghdad-Iraq.
• There was an increased frequency of HLADRB1*03:01:01 in control group compared with patients group (P = 0.009, Odds ratio = 0.1228, 95% CI: 0.0254–0.5930). Other allele like HLA-DRB1* 070101 was significantly increased in asthmatic patients in compares with control group (P = 0.005, Odds ratio = 6.641, 95% CI: 1.7319–25.4657).
• HLA alleles have an effect on development asthma in patients with HLA-DRB1*070101 while HLADRB1* 030101 may have a protective effect.

## 1 Introduction

Asthma is one of chronic inflammatory diseases of the airways that affect individuals at any age. It induces recurrent episodes of wheezing, cough, chest tightness and shortness of breath. The airways are chronically inflamed and obstructed by contraction smooth muscle around airways, making the air way smaller and thick mucus plug it [1]. The inflammation is due to infiltration Th2 lymphocytes, mast cell and eosinophils. Th2 lymphocytes secrete cytokines that play important role in orchestrating the inflammatory responses [2]. In addition to that, Th17 lymphocytes have been involved in the pathogenesis of asthma [3].

Asthma is a complex obstructive respiratory disease that is caused by the interaction of genetic susceptibility with environmental influences. Genome-wide screening studies have identified many chromosomal regions containing susceptibility genes for asthma such as 2q, 3p, 5q, 6p21 and 12q23 [4]. Human Leukocytes Antigens (HLA) located on chromosome 6p21 are among the most polymorphic genes, which is associated with other aspects of immune response, some complement components, and susceptibility to certain diseases due to variations of the peptide binding cleft influencing the bound antigens and presented to T lymphocytes [5]. In general, HLA class I molecules present foreign antigens that binds the cleft to T CD8^+^ cytotoxic T lymphocytes and HLA Class II molecules present foreign antigenic peptides to CD4^+^ T helper cells [6]. HLA plays a critical role in human immunological diseases, transplantation, host defense against infections, and all known risk factors for asthma [7]. However, the crucial roles of the HLA system for regulating the susceptibility to asthma become more clear [8]. Researches demonstrated the relation of HLA- DQB1 and HLA- DRB1 alleles with asthma. Morris et al., 1977 [9] reported that HLA- B8 was associated with asthma while Movahedi et al., 2008 [10] showed that HLA-DRB1*12, DQA1*0505 & DQB1*0603, *0604 alleles might be associated with asthma while Turton CWG. et al., 1979 [11]demonstrated not such relation with asthma due to different race group. Therefore, different studies have shown that different HLA alleles are associated with a population of asthma. The aim of the present study was to evaluate the association of HLA genes with asthma in Iraqi patients.

## 2 Patients and methods

Forty unrelated patients with asthma (20 males, 20 females, mean age 36.5 ± 0.2 years with range 23–58 years), referred to Medical city Teaching Hospital at Baghdad University were enrolled in this study. All of the patients were diagnosed based on the definition of American Thoracic Society including clinical symptoms of asthma like wheezing, cough, chest tightness, dyspnea relieved by an inhalation β-agonist drug and corticosteroids according to guidelines for the diagnosis and management of asthma and did not classified according to their severity of symptoms [12].

Forty normal blood donors (20 males, 20 females) ethnically and age matched with patients group with no history of atopy, asthma and allergic diseases were randomly selected as control group. The mean age of the control group was 36.4 ± 0.2 years with range 23–58 years.

The Scientific and Ethical Committee of Al-kindy medical college and Medical City Hospital had approved the study. Written informed consents were obtained from the patients with asthma and control normal blood donors.

### 2.1 HLA class II typing (DRB1)

Five mL of venous blood were collected by venipuncture from study and control population in EDTA containers for DNA extraction by blood kit (QIAmp DNA blood Mini Kit, QIAGEN INC- Germany). DNA product was verified by electrophoresis in a 2% agarose gel containing ethidium bromide and was visualized under UV light. Locus- and allele-specific amplification of genomic DNA was performed for DRB1. Amplification and Hybridization was performed using a panel of sequence-specific oligonucleotide probes (SSOP) using HLA-DRB1amplification and hybridization kits (SSO HLA type DRB1 plus and Mastermix for HLA type DRB1 Amp plus kits -Innogenetics-Belgium) using automated method by AutoLipa – 48Innogenetics-Belgum. The results were interpreted using LiRas version-5.0 software- Innogenetics-Belgium.

### 2.2 Statistical analysis

HLA frequencies were determined by direct counting. The frequency of each allele was compared between asthmatics and normal control group using chi-square test fisher exact test using MiniTab version. 3.0 software. In each comparison, the Odds ratio (OR) along with the 95% confidence interval (95% CI) was used. P-value less than 0.05 were considered statistically significant.

### 2.3 Strocss

The work has been reported in line with STROCSS criteria and site the paper as follows:

Mahdi BM, Al-Hadithi ATR, Raouf H, Zalzala HH, Abid LA and Nehad Z, Effect of HLA on development of asthma.

## 3 Results

A total of 40 patients with asthma were enrolled in this study. 20 (50%) of the patients were females, 20 (50%) were males, with ratio 1:1 males to females. Mean age was 36.5 ± 0.2 years with range 23–58 years. The other group was forty normal blood donors (20 males, 20 females), their ages were 36.4 ± 0.2 years with range 23–58 years (Table-1).

**Table-1:** Demographic data of asthmatic patients.

Parameters	Asthmatic patients No. = 40	Control group No. = 40	P -value
Sex (M/F)	20/20	20/20	NS
Age (years) X±SD	36.5 ± 0.2	36.5 ± 0.2	NS
Smoking No.(%)	35(87.5)	1(2.5%)	0.000

The distribution of HLA polymorphism HLA*DRB1 was investigated in the control and patients groups of Iraqi Arab Muslims. The observed and expected phenotypes of all loci for the control group as demonstrated in Table 2 were in a good agreement with Hardy-Weinberg equilibrium as shown in Table 3.

**Table-2:** Observed and expected numbers, percentages of HLA*DRB1 in healthy control Iraqi population.

HLA-DRB1* alleles	Healthy control group No. = 40 Observed		Healthy control group No. = 40 Expected	
	No.	%	No.	%
02:03:01	3	7.5	2.33	5.84
03:01:01	12	30	11.68	29.2
03:01:02	1	2.5	0.94	2.36
03:06:01	18	45	16.99	42.47
04:02:01	0	0	0	0
04:03:02	1	2.5	0.94	2.36
04:04:01	2	5	1.56	3.91
07:01:01	3	7.5	2.33	5.84
07:07:01	1	2.5	0.94	2.36
08:07:01	3	7.5	2.33	5.84
11:01:01	7	17.5	6.93	17.33
11:02:01	3	7.5	2.33	5.84
11:03:01	5	12.5	4.60	11.51
11:05:01	0	0	0	0
11:16:01	5	12.5	4.60	11.51
11:37:01	0	0	0	0
12:09:01	4	10	3.86	9.65
13:01:01	0	0	0	0
13:59:01	3	7.5	2.33	5.84
14:01:01	3	7.5	2.33	5.84
14:33:01	3	7.5	2.33	5.84
15:01:01	3	7.5	2.33	5.84
16:01:01	0	0	0	0

**Table-3:** Hardy-Weinberg equilibrium in HLA*DRB1 in healthy control group of Iraqi population.

population	Locus	X^2^	D.F.	P
Healthy Iraqi control group	HLA*DRB1	1.794	22	NS

DF = degree of freedom.

NS=Not significant.

Control and asthmatic patients were typed for identifying the DRB1* alleles using DNA based methodology (PCR-SSOP). There was an increased frequency of HLADRB1*03:01:01 in control group compared with patients group (P = 0.009, Odds ratio = 0.1228, 95% CI: 0.0254–0.5930). Other allele like HLA-DRB1* 070101was significantly increased in asthmatic patients in compares with control group (P = 0.005, Odds ratio = 6.641, 95% CI: 1.7319–25.4657) as shown in Table 4.

**Table-4:** Human leukocytes antigens (HLA-DRB1) alleles frequencies in patients with asthma and healthy control groups.

HLA-DRB1* alleles	Asthmatic patients group No. = 40		Healthy control group No. = 40		Odd ratio (95% confidence interval)	P- value
	No.	%	No.	%		
02:03:01	0	0	3	7.5	na	na
03:01:01	2	5	12	30	0.1228 0.0254–0.5930	0.009
03:01:02	2	5	1	2.5	2.052 0.178–23.590	0.563
03:06:01	20	50	18	45	1.222 0.507–2.943	0.654
04:02:01	4	10	0	0	na	na
04:03:02	4	10	1	2.5	4.333 0.462–40.610	0.199
04:04:01	0	0	2	5	na	na
07:01:01	14	35	3	7.5	6.641 1.731–25.465	0.005
07:07:01	2	5	1	2.5	2.052 0.178–23.590	0.563
08:07:01	2	5	3	7.5	0.649 0.102–4.110	0.646
11:01:01	4	10	7	17.5	0.523 0.140–1.953	0.335
11:02:01	0	0	3	7.5	na	na
11:03:01	0	0	5	12.5	na	na
11:05:01	2	5	0	0	na	na
11:16:01	0	0	5	12.5	na	na
11:37:01	2	5	0	0	na	na
12:09:01	0	0	4	10	na	na
13:01:01	10	25	0	0	na	na
13:59:01	2	5	3	7.5	0.649 0.102–4.110	0.646
14:01:01	4	10	3	7.5	1.370 0.286–6.55	0.693
14:33:01	2	5	3	7.5	0.649 0.102–4.110	0.646
15:01:01	2	5	3	7.5	0.649 0.102–4.110	0.646
16:01:01	2	5	0	0	na	na

na = not applicable.

## 4 Discussion

There are numerous genome-wide studies showing the importance of genetic factors in development of asthma such as those located in 2q, 3p, 5q, 6p21 and 12q23 chromosomal regions [13]. After description of serological and different molecular techniques for HLA typing, studies on the association of the HLA allele that located on 6p21 and the susceptibility to or protection against asthma started. In our research, we investigated the association of HLA class II (HLA-DRB1) with asthma in Iraqi patients. In our study, the HLA-DRB1* 07:01:01 was significantly increased in asthmatic patients in compare with control group (P = 0.005, Odds ratio = 6.641, 95% CI: 1.7319–25.4657), whereas HLADRB1*03:01:01 allele showed decrease in asthmatic patients (P = 0.009, Odds ratio = 0.1228, 95% CI: 0.0254–0.5930). Turton et al., 1979 [14], found no association between asthma and HLA or with total serum IgE concentration. While other study by Movahedi et al., 2008 [15] demonstrated that HLA-DRB1*12 significantly increased in asthmatic patients and HLA-DQB1*0603 and 0604 alleles were significantly higher in asthmatics than those in normal controls. Conversely, HLA-DQB1*0501 and 0602 were decreased in asthmatics compared to normal controls. The most frequent alleles in asthmatic patients with the total IgE>200 IU/mL were HLA-DRB1*11 and 1401, HLA-DQA1*0505, HLA-DQB1*0301 and in patients with total IgE<200 IU/mL were HLA-DRB1*0301, 07 and 1301, HLADQA1*0201 and 0301, HLA-DQB1*0201. These data suggests that HLA-DRB1, DQA1 & DQB1 alleles and haplotypes might be implicated in susceptibility to allergy and asthma and serum IgE production. In other study by Gao et al. (2003) [16] that done in china, HLA-DQA1*0104 and -DQB1*0201 were positively associated with asthma while HLA-DQA1*0301 and -DQB1*0301 alleles were negatively associated with asthma. The study by Parapanissiou E. et al. [17], in Greek children with allergic asthma revealed that DRB1*04 and DQA1*0301 might be important factors in susceptibility to asthma with sensitivity to mites while a Korean study reported the higher frequency of HLA-DRB1*07 in mite sensitive asthmatics than in control group(17). This difference may be attributed to some genetic impact on the specific allergen sensitive asthma and to some degree to the genetic and race population differences [18]. Other study in Thia reported that HLA-DRB*1454 is a predictor of new asthma in HIV infected children [19]. In India [20] and USA [21], studies showed a significantly higher frequency of HLA-DRB1*03 in asthmatics than in controls while in our study we found HLA-DRB1*03 is a protective allele against asthma. This discrepancy is due to race or ethnicity, genetic factor, sample size, method used in the study, type of allergen that exposed to it, smoking, type of exposure to environmental factors and age of onset of asthma. Mubarak B et al., 2017 [22]showed HLA-DQβ1*0201 and -DQβ1*0301 alleles were not associated with asthma; however, HLA-DQβ1*0201 allele was associated with low levels of total serum IgE in the serum. In addition to that, a Canadian study confirms that HLA is a risk factor for peanut allergy independent of asthma [23]. Other study stratified the study group according to age and revealed that DRB1*03, DRB1*04, DRB1*09, and DRB1*15 were associated with asthma in children. Subgroup analysis by ethnicity showed that DRB1*03 and DRB1*15 were associated with asthma in whites, and DRB1*07 and DRB1*14 were associated with asthma in Asians [24].

In conclusion, it is suggested that HLADRB1*0701 and HLADRB1*0301 alleles might be the possible genetic factors for susceptibility and protection against asthma respectively. This is the first study showing HLA allele association with asthma in Iraqi population. Future studies are needed to investigate the association of HLA alleles in patients with allergic asthma to certain environmental allergens. Also, other identified HLA alleles associated with total IgE level should be confirmed through another study using allergen-specific IgE level. There is a limitation to this study like small number size of the patients' sample and studying other alleles of other locus. So the suggestion is include larger number of the asthmatic population and sorted them according to age like childhood asthma and adult asthma.

## 5 Conclusions

HLA alleles have an effect on development asthma in patients with HLA-DRB1*070101 while HLADRB1* 030101 is a protective allele in Iraqi Arab Muslims individuals against development of asthma.

## Ethical approval

Yes.

## Funding

Iraqi Research and development department- Ministry of Higher Education and Scientific Research. University of Baghdad and Al-Kindy College of Medicine.

## Author contribution

Dr. Batool wrote paper.

Dr Arwa collected samples.

Dr Hyam did tests.

Dr Haider did tests.

Laheeb and Zena did tests.

## Conflicts of interest

The authors have declared that no competing interest exists.

## Research registration number

UIN = 4079.

## Guarantor

Consultant Dr Ali Ghalib Mutar Mahdi.

## Provenance and peer review

Not commissioned, externally peer reviewed.
